# Selections

**Published:** 1849-01-01

**Authors:** 


### Selections.

The Pulse of the Insane.—M. Jacobi has instituted experiments on the pulse in a great many cases of the different forms of insanity, and also on persons in good health, in the hope of being able to draw conclusions with reference to the relation of the circulation with the different pathological states of the intellect and the feelings. The experiments were performed on upwards of 350 persons. Three times a day, at six, A.M., at two, P.M., and at eight, P.M., the number of pulsations was ascertained, and a vast number of meteorological and physiological observations noticed. He was not contented, however, with merely ascertaining the frequency of the pulse, but he took an exact note of its greater or less degree of strength, of fulness, of slowness, or of tension, in the insane patients who were under observation. He indicated, besides, the relative pulsations of the different arteries, auscultated the heart, and counted the number of inspirations and expirations; the state of the iris even was examined, with reference to the general circulation; nevertheless, he remarks, "I had the vexation to see that my researches, so conscientiously made, did not fulfil the end I had proposed; and I saw that it was impossible to establish the necessary connexion between the different pathological states of the intellect and feelings, and the observations I had collected on the state of the circulation, the respiration, and the temperature of the skin, in the insane."—*Annales Medico-Psychologiques.*

Compression of the Carotids in Insanity.—The phenomena noticed by M. Jacobi, as resulting from this operation, were very variable; the principal were, a sensation of burning heat, which extended over the whole head and neck, and even to the chest. Others complained of the same sensation inside the head. The sight was affected in many persons, and brilliant substances seemed to pass before the eyes. It was not uncommon for them to complain of great difficulty of breathing, amounting even to agony. This was accompanied by a sensation of weight in the head, giddiness, and a tendency to sleep, which, in some cases, really occurred, the breathing being stertorous. It is probable that in these cases sensation was lost, although it is not mentioned by Jacobi. Syncope was of common occurrence; and when not complete, the patients complained of a difficulty in moving the lower extremities. One person, during the compression of the carotids, fell as if struck by lightning; but he soon got up again, and did not experience any further ill consequence. In another person, the syncope was of considerable duration.

M. Jacobi mentions a phenomenon which has not been described by Burrows or Parry, namely, the sudden diminution in the beats of the radial artery, in consequence of compression of the carotids. In seven-eighths of his patients, the pulse fell from 100 to 66—from 72 to 48. In three instances only the frequency of the pulse was increased.

Those persons are more readily affected by carotid compression, in whose temperament the venous circulatory system predominates, or whose cerebral activity has been increased by immoderate indulgence in sexual gratification, or alcoholic drinks. Adults in the prime of life are more easily brought under the influence of this operation than women, children, or old men. It appears to be injurious in cases of cerebral congestion, probably owing to the then existing greater preponderance of the venous circulation. It is, therefore, an operation requiring peculiar precautions, and one that it would be dangerous to employ indiscriminately.—*Ibid.*

Treatment of Epilepsy.—M. Delasiauve, in the concluding portion of his essay on the treatment of epilepsy, directs especial attention to the importance of hygiene, which he considers to equal, if not surpass, that of medicine in the disease. There are some practitioners, even, who look upon medicine as utterly useless in such cases, and place their sole reliance on such measures as serve to guard the patients against the causes which induce the fits, and favour the action of such natural agents as are capable of changing the constitution. Hippocrates recommended a change of climate; and Van Swieten mentions several instances in which epileptic patients were freed from their fits all the while they remained in the East Indies. M. Delasiauve advises, when a change of climate is practicable, that a gentle, temperate climate should be selected—one but little subject to atmospheric changes; because experience has shown, in those asyla where epileptic patients are admitted, that the fits are much more frequent during extreme cold or extreme heat, and especially during continued variations of temperature. With respect to diet, the temperament, idiosyncrasy of the patient, state of the constitution, and his usual habits of living, will more or less modify it; but nevertheless it may be stated, that excess in quantity or quality, of either food or drink, will prove injurious. Every infraction of the rules of temperance will induce a relapse. More vegetable than animal food should be taken, and cooling fruits may also be used. Complete abstinence from wine is perhaps hardly necessary; but if it be drank, the wine selected should be the least stimulant, and even then only taken in moderation. Those patients who are liable to be attacked during the night, should make but a light supper, to avoid increasing the cerebral plethora, which is always greater during sleep. The necessity of maintaining the excretions must be self-evident. With respect to the insensible perspiration, cleanliness, baths, pediluvia, frictions, and warm clothing are requisite. Hard cravats and stays are decidedly injurious; and straw hats are better than the hats and caps which are in common use. The hair should be cut short; in bed, the patient should lie with his head high, to assist the circulation of the blood through the brain. Constipation should be avoided; when it occurs, it must be treated by injections and laxatives; the digestive organs should be especially attended to.

A disordered condition of the menstrual secretion is generally the cause of increased severity of the fits. The ordinary recurrence of the discharge is frequently sufficient to induce a fit, so that the attention of the practitioner should be directed to this secretion, to maintain it in a healthy state.

Continence is essentially the virtue of the epileptic; sexual intercourse produces a nervous shock, which too closely resembles the emotion which occasions the epileptic attacks, not to be attended with great danger. Those who practise onanism have, in general, the greatest number of fits. A peaceable and quiet life suits the epileptics best. Everything that tends to excite their feelings, to rouse their passions—the strong feelings of love, contradiction, and grief, inevitably add to the intensity of the disease. Their situation demands the greatest management, as they are in general very susceptible and irritable, especially just before and after a fit. Exercise is very salutary; an inactive, sedentary life, increases the morbid predisposition, and renders the consequences of the fits more deplorable. Hippocrates and Galen lay great stress on bodily exercise. Esquirol recommends gardening, horse-exercise, the gymnasium, swimming, fencing, &c. Some of these M. Delasiauve considers objectionable. M. Ferrus, on whose opinion our author evidently places great reliance, depends much on the utility of out-door work, such as agriculture and gardening. While acting as the principal physician at the Bicêtre, M. Ferrus tried this plan of treatment somewhat extensively, both with the insane and with epileptic patients. Both classes were benefited, but the latter most so. The utility of gardening is equally discoverable in private life. Of this we have in the essay before us an illustration, in the case of a gentleman subject to epileptic fits, for which medical aid was powerless, but which were entirely arrested by a journey to Switzerland, and garden-work, carried on by the patient for a series of years. Other cases are also alluded to, in which a similar result followed the having recourse to garden employment.

Horse exercise, swimming, and fencing, which, as previously mentioned, are recommended by Esquirol, are stated by M. Delasiauve to be dangerous, as are also all exertions which require too large an expenditure of strength, or a stooping posture, or in which the body is exposed to a very intense heat or light.

Variety of occupation, intermingled with amusing relaxations, will prove serviceable in cases of epilepsy. Intellectual employment requiring deep thought is injurious. Reading, drawing, music, light compositions, and the elements of chemistry, botany, physics, &c., afford great satisfaction, and sustain the moral powers, instead of exhausting them.

In his concluding remarks, M. Delasiauve recommends the establishment of institutions for the reception of epileptic patients, in order that an hygienic as well as a medical plan of treatment may be carried out, as is practised at the Bicêtre. He considers them to be absolutely indispensable.

With respect to the treatment during the fit, it resolves itself almost entirely to the prevention of bodily danger by falls or otherwise. The patient should generally be placed on his back in bed, all tight articles of clothing removed, the head a little raised by pillows, in order to diminish the determination of blood to the head, and the body placed a little on one side, in order to favour the discharge of saliva, which collects in quantity in the mouth, and might otherwise prevent the passage of air into the lungs. Some patients, when attacked during the night, have an unfortunate tendency to turn on the face, and unless carefully watched, and their position changed, may die asphyxiated. Another accident, which occurs in some instances, is the laceration, or even the amputation, of the tongue during the fit. To prevent this, a piece of wood, or a linen roll, may be placed between the teeth when the fit is coming on.

The symptoms consecutive to the attack occasionally require the attention of the medical practitioner. Generally speaking, the patient complains simply of a little fatigue, heaviness, and headach, which may be removed by rest, or by a slightly sedative or cordial infusion, with sinapised pediluvia. In the more severe cases, where the symptoms indicate congestion of the brain or lungs, the indication of practice is the abstraction of blood by large general bleedings, assisted by cupping and the application of leeches. The loss of blood is readily borne under such circumstances. Warm baths, external revulsives, sinapisms, flying blisters, &c., may also be had recourse to. The utility of refrigerant applications appears to M. Delasiauve to be problematical.—*Annales Medico-Psychologiques.*

Lunatic Asylum, Nantes.—Patients are placed in the lunatic asylum at Nantes, either by their friends or by order of the authorities. The former is entitled *placements volontaires,* a term that does not indicate that the patient has entered of his own free will, although such examples occasionally occur, but that his friends have placed him there on the authority of a medical certificate, which should indicate his mental condition, the symptoms of his disease, and the necessity of treating his case in an asylum, and of keeping him in one. One of the persons thus admitted was a patient there for the second time. His insanity consisted, not in a delirium of words, but of actions; such as sensual appetites, a desire for continual motion, and the purchase of all sorts of articles without utility. M. Verdon, the physician to the asylum, certified that he was insane—that the disease affected the will and the character, rather than the ideas and the judgment, although the reason could not be said to be sound—that the insanity was characterized by a tendency to excesses of all kinds, to extravagant expenditure, and romantic and ridiculous eccentricities. M. Foville, of the Charenton, fully agreed with M. Verdon, that the patient was a fit person to be admitted into the asylum; but notwithstanding, on the perusal of their certificates, and the reading of a letter written by the person, he was set at liberty by the authorities. The reporter observes on this case, that justice is always unwilling to consider a man insane who can write a reasonable letter, and sustain a long conversation without any wandering in his ideas. Extraordinary actions, excesses, extravagant expenditure, and romantic and ridiculous eccentricities, it refers to a bad education and pernicious habits.

The difficulty of this limitation, however, says M. Bouchet, the reporter, which equally exists for the disease of the intellectual faculties, ought not to prevent our acknowledging this species of insanity, which, without affecting the intellect, acts more especially on the moral principles. Psychologists have distinguished three distinct groups of the faculties of the soul—sensibility, intellect, and the will: sensibility, which comprises the pleasures and the pains, the appetites, the desires, and the more or less reflective tendencies, that is, the sentiments; the intellect, that is, the ideas, the notions, the thoughts; and the will, which comprises the determinations and the resolutions, in a word, action. This latter faculty is the result of the coöperation of the other two, or of one of the two. Sensibility, intellect, and the will decompose in the psychological as in the material order, and act together, or are separated more or less completely in the pathological order. In order that man may be in the full possession of reason, there must be a simultaneous action of these three faculties in their regular or habitual course; but if, under certain organic dispositions produced by disease, the equilibrium is lost between these different forces, the mental faculties, which comprise the moral as well as the intellectual feelings, are changed in their manifestations, and mental alienation is the result. The conscience itself can no longer prevent the wanderings of the faculties; influenced by internal sensations, it seeks its impressions in the deposed notions and recollections of the intellect, as well as in the deposed feelings of sensibility. With these elements it judges actions; thus it justifies murder with Brutus and Charlotte Corday, suicide with Cato, and persecution with Philip the Second and Robespierre. The lunatic who has a knowledge of the morality of his actions, without the power of controlling them, or preventing their wanderings, judges and thinks rightly, but he feels wrongly; and this perverted feeling, superexcited by disease, commands the will, which directs the action in spite of his judgment, the healthy state of which has rendered it powerless.

It is not therefore necessary that a patient should wander in his ideas, judgment, reasoning, and language, in order that he be declared insane; it is enough that he shows it by eccentric and abandoned actions, caused by a diseased imagination, which the judgment is incapable of controlling. If, in the sequestration of the insane, a separation should be made between those whose insanity was indicated by their words, and those who evinced it by their actions, there would be but little room for hesitation. The latter ought to be submitted to sequestration rather than the others, because perversions of the sensibility cause more injury in social life than the perversions of the intellect.

In dementia, the manifestations of insanity are less evident, because all the faculties are merely weakened, (*affaiblis.*) The disorder often ceases, as regards words and actions, but the patient speaks and does little; he replies at greater length than he questions, and his actions are more routine than voluntary. The manifestations of delirium take place only under some excitement influencing the intellect or the sensibility, which gradually become weaker, so as, at last, not to be influenced by any excitant. One can do as one will with these patients, except induce them to reason. They have lost the power of combination. When excited by more or less vivid impressions, the disorder returns, and its manifestations are often dangerous. Of this kind, two or three examples are given in the report. In one of these, the patient, a lawyer, having left an asylum apparently convalescent, while travelling in Switzerland, mixed with society, and also bathed in the sea. The result was, that in a few days, mental delirium was displayed in the form of all kinds of eccentricities, so that he was again placed in an asylum, labouring under insanity and general paralysis, and died, some months afterwards, at Charenton.

The next case exhibits, in a painful manner, the consequences of setting at liberty a man labouring under dementia. The patient was an old advocate; his complaint was demency, with general paralysis; his idea, the extent of his riches. He was placed in the asylum, and an interdiction obtained with respect to his property. Becoming apparently better, he was removed; relapsing, he was admitted into another asylum, whence he was withdrawn, on again improving. He then obtained the withdrawal of the interdiction, and at once plunged into a wild career of speculation, making also ridiculous purchases, until he had ruined his own fortune, and that of his children, when the disease carried him off.

With respect to the sequestration of the insane in asyla, it appears that in France a law passed in 1838, (article 8,) requires the medical man to state in his certificate, not merely the facts of the insanity, but also that the patient's sequestration is necessary, otherwise he cannot be received, unless the insanity be of a character to interfere with the public welfare, or to endanger the lives of individuals. M. Bouchet dwells somewhat on the difficulty of indicating precisely the characters of insanity which render a patient dangerous, and require him to be sequestered. He considers those persons who, in their insanity, offend against public order by wild cries, gesticulations, and menaces, and by causing alarm,—the religious, sombre enthusiast, who fancies he does God service by committing murder,—and, again, the man who fancies himself pursued and persecuted by imaginary enemies, to be among those who require sequestration; but the patient labouring under dementia, and the quiet idiot, may be, he thinks, kept under the control of their respective families. Occasionally, he admits, they may revive from their apathy, and cause some annoyance to society, but this, he says, is but momentary, and not of a nature to necessitate sequestration.

The withdrawal of patients from the asylum is effected, first, in consequence of a cure; secondly, by the desire of the friends; thirdly, by order of the prefect; fourthly, by order of the tribunal; fifthly, by escape; and sixthly, by death.

In some cases, the indications of the cure are readily perceptible, but in those instances in which the disease consists of an exaggeration to delirium of an habitual state of intellect and sensibility, disordered by nature or a bad education, the signs are less perceptible, and the patient requires to be watched after he has left the asylum. This precautionary measure is recommended in all cases. In some cases of insanity the lucid intervals are so well-marked that the patients, although not cured, may yet enjoy their liberty. In other, but very rare instances, the dismissal of the patient before a cure has been effected becomes a necessity, in order not to cause by a further sequestration an aggravation of the disease. This was exemplified in the case of a young woman, whose mind was impressed with the belief that her family were all in the asylum. Her dismissal took place, and in a short time she was convalescent.

The second mode by which patients are withdrawn from the asylum, is by the interference of the friends against the wish of the physician. Of the injurious effects of this proceeding two instances are narrated: the second is the more painful of the two. A lady, forty years of age, labouring under incurable dementia, was withdrawn by her mother and brother, in spite of the earnest opposition of the physician, who yielded only on the promise of the brother that she should be under his surveillance. This promise, however, was broken, and the old lady was left alone with her insane daughter. The consequence was, that the latter murdered her mother, by separating her head from her body, and was then taken back to the asylum, which she ought never to have left.

The patients who are dismissed by order of the prefect are those whose mental state is such as, under certain conditions, is compatible with the arrangements of social life.

The fourth mode of withdrawal of a patient, by order of the tribunal, is a legal process, and one not unattended with bad consequences.

The fifth is by evasion. This is partly attributed by M. Bouchet to the insufficiency of the keepers, who, in consequence of the paucity of the pay, are generally persons whose capacity unfits them for other employment. He mentions an interesting fact, that in consequence of a new superintendent (*surveillant du personnel*) having been appointed, ten escapes took place, he not having had time to make himself acquainted with the persons under his charge. Another cause which leads to escape is the insufficiency of the buildings where the lunatics are confined, more especially in the places where they work. As in the latter case only quiet patients can escape, no great injury, M. Bouchet thinks, can accrue, if they are soon re-captured; but it is different when the escape takes place from the asylum, where the strictest precautions should be enforced, and the building be so constructed as to prevent escapes. One lunatic has escaped five times from the asylum, and four times from the Bicêtre; and another, a homicide, made his escape with the strait-waistcoat on.

The sixth mode, that of death, may be the result of violence or accident, or from the mental disease itself, or the accidental diseases by which it may be complicated. The acts of violence may be committed by the keeper in a fit of anger against the lunatic, induced by an assault against him. To avoid this as much as possible, the service is so arranged that the keepers are seldom alone, so as to have an imposing array of strength to overcome the violent lunatic. They also have an apron, which they are instructed to throw over the turbulent patient's head, so that he can no longer see where to direct his blows. The keepers are authorized to put on the strait-waistcoat; but confinement in a cell can only take place by order of the physician. Accidents occur to the insane while at work, as they do to other workmen, and from the direct violence of the patients, while labouring under delirium. Of the former only two have proved fatal in fourteen years. Suicide is rare; but attempts at self-murder are frequent. The refusal to take food is generally obviated by the use of the œsophageal sound, modified by Leuret and Baillarger. Generally speaking, this plan soon succeeds in overcoming the refusal; but one patient persisted for several months, alleging that her food was poisoned. She died of gangrene in different parts of the body. Hanging is a mode of self-destruction frequently attempted. One patient succeeded in destroying himself by tearing the strait-waistcoat, and strangling himself with one of the bands. Another patient poisoned himself with glass, which he had managed to collect and break up; and another threw himself under a cart-wheel, but without experiencing any injury.

Death, as the direct result of insanity, occurs but rarely in the asylum. M. Bouchet refers the mortality of the insane to acute or chronic cerebritis, the latter showing itself in the form of dementia, complicated with general paralysis. The mortality in 1846 was nineteen per cent. among the men, three per cent. among the women. This was owing to the greater number of cases of dementia, a disease more prevalent among men than women. Among the diseases which complicated insanity was scurvy, which showed itself in several cases of dementia; these terminated fatally. It also appeared in patients affected with other forms of insanity. Of these latter, two died, and about from seventeen to twenty recovered. The only cause that M. Bouchet can assign for its occurrence, is the inertia which attends dementia.—*Annales Medico-Psychologiques.*

Attempt at Murder: Insanity.—A man named Miguand, *alias* L'Oreille, *alias* Alexis, was, in December, 1847, tried at the Court of Assizes of the Seine, for attempting to murder the police-serjeant, who was about to arrest him for breaking his ban. Certain facts having come to the knowledge of the magistracy, they directed an inquiry to be made as to the state of his mind. Dr. Jacquemin, physician to La Force, reported that "Miguand is a complex being: in his ordinary state he is gentle, and as easy to lead as a child; but when opposed, he becomes ferocious, and resembles a brute beast. On one occasion he literally crushed a tumbler between his teeth. His excitement is such, that, in order to restrain him, it is necessary to put irons on his hands and feet." Dr. Jacquemin's opinion, however, was, that the accused fully understood the nature of the crime he had committed. The crown eventually abandoned the prosecution on the production of a certificate from the physician to the Madelonnettes, stating that, in 1838, the prisoner was confined there for confirmed insanity. The jury returned a verdict accordingly.

A few days afterwards, Miguand was brought before the tribunal of Correctional Police in Paris, on the charge of breaking his ban. His costume on that occasion was *bizarre.* On his prisoner's dress he wore the grand cross of the Legion of Honour; at his button-hole there was a large pasteboard cross; from his neck there hung a myriad of amulets, &c., and his fingers were loaded with rings. The contradictory nature of the reports from La Force and the Madelonnettes induced the court to order a fresh inquiry to be made into the state of the prisoner's intellect.

Infanticide.—Louis Cellier, a priest, serving the parish of St. Gall, in the diocese of Clermont, was arraigned at the Cour d'Assises du Puy de Dôme, on a charge of infanticide, the victim being his illegitimate offspring, by his servant. The plea of insanity was alleged in his defence. Several of the witnesses gave it as their opinion that *s'il n'était pas fou, il était bien drôle tout le même.* During the mission he himself rang the bells at all hours of the night. One day, during the procession, he got a drum, and began to beat it; he did the same on another occasion in the sacristy, while they were singing. Again, when returning from administering the viaticum, and passing through the village, he shouted out the song, "*Reveillez vous, belle endormie.*" He used to consecrate eight hundred hosts for the spiritual wants of the inhabitants of the commune, although they were only three hundred in number. When he was the vicar at Maraingues, he used to get up at night, and go out alone, or accompanied by a man he employed, and, armed with a poniard, sword, or other weapon, give way to the most extraordinary courses in the fields; or else, suddenly seized with a panic fear, would flee until overcome with fatigue. At other times, he would turn his weapons against his companion, and even against a tree, which he would combat with, until he fell from exhaustion. His mother was insane from an early age.

Louis Cellier was found guilty of the crime of infanticide, with mitigating circumstances, and sentenced to five years' hard labour. The sentence did not change the expression of the prisoner's features in the least—they bore the same pale and suffering imprint as before, the same vague and unintelligent look.—*Gazette des Tribunaux.*

In this case, according to the editor of the *Annales Medico-Psychologiques*, the medical men who were consulted considered Cellier's conduct to indicate great excitement and *bizarrerie*, but not to amount to mania. The editor of the journal observes that the court ought not to have been content with this opinion, but to have summoned medical men who have devoted themselves to psychological studies, as they alone are competent to judge of the mental state of an accused person. However enlightened and well-informed they may be, other practitioners are not able to seize the more minute indications of insanity, since even psychologists themselves in some cases require a great deal of time and attention to declare the presence or absence of insanity.

Cerebral and Meningeal Phlebitis in Puerperal Women.—M. Ducrest, who has published an essay on the occurrence of cerebral and meningeal phlebitis in puerperal women, states that the disease is met with but rarely. When it is met with, however, it constitutes a very dangerous complication of the puerperal state. Encephalic phlebitis presents all the characters which belong to the anatomical lesions observed in the phlebitis of other organs, that is to say, it is adhesive or suppurative. In the first case, we find, in the calibre of the cerebral vessels, a firm clot adhering to the vascular parietes; in the second, pus is found in the veins and in the cerebral mass, either infiltrated or in the form of abscesses; the extent, seat, and number, may vary. As the result of these lesions, or occurring at the same time, we may note other secondary alterations, such as meningitis, meningo-encephalitis, hemorrhage, and ramollissement. In all cases there were purulent collections elsewhere besides the brain, that is, in the uterus, lungs, &c. It is worthy of notice, that cerebral phlebitis principally attacks tuberculous subjects; M. Ducrest looks upon it as a secondary disease, a consecutive phlebitis.

The symptoms described by M. Ducrest are chiefly those which are indicative of cerebral mischief; they are not diagnostic of the disease in question. They are—more or less intense headache, delirium of a variable character and duration, preceded or followed in some cases by a sort of obtuseness or torpor of the intellect; convulsions, trembling of the muscles of the limbs and face; eclampsia, palsy, generally partial and incomplete; contraction, generally backwards, even to convulsions; numbness of the limbs; feeling of burning and weight in the eyes, irregular dilatation of the pupils, &c. These symptoms are neither constant nor diagnostic; nor are they certain data for the prognosis and treatment of the disease. M. Ducrest nevertheless concludes, that the occurrence of all or of the greater part of these symptoms indicates an organic affection of the brain, whatever it may be, which consequently ought to attract the attention of the practitioner; that it should be treated according to the strength of the patient, and the therapeutic rules which guide the treatment in acute diseases of the brain; and lastly, that, as there are stages in the progress of encephalic phlebitis, a prompt and active treatment should be adopted in the early part of the disease, to prevent its passing into the suppurative stage.—*Archives Génèrales de Médecine.*

Treatment of Epilepsy.—M. Mettais, at Montrouge, strongly recommends the employment of tartarized antimony externally in frictions on the head, in cases of epilepsy. He mentions several cases in which benefit has been derived from this plan of treatment, which is not, however, a novel one, counteraction having been employed long since, although now much neglected.—*Gazette Médicale.*

M. Le Breton describes, in another number of the same journal, a case in which he had recourse successfully to a much more severe treatment for the cure of this disease. He was consulted by a young man, who had epileptic fits daily, and whose countenance bore an impression of hebetude. M. Le Breton applied the actual cautery, two lines in diameter, to the sinciput, keeping the instrument applied about twenty-five seconds, and pressing so as not to affect the whole thickness of the skin. The cautery was applied once a week for about three weeks, during the whole of which time the patient did not have one fit. The cautery was not then applied until the twelfth day, and then more superficially than before. M. Le Breton saw the patient a fortnight afterwards, quite cured, his intellect and strength being fully restored.

The same state of good health persisted when the patient was seen by M. Le Breton about three months afterwards.

Nocturnal Neuralgia of the Forearm.—M. Gambèrini, in describing this singular disease, states that it commences by a pain at the end of the fingers of one hand, generally the two last, and then extends along the forearm, to within one or two inches of the elbow. The pain has always stopped there in the cases which have fallen under M. Gambèrini's notice. The night is the time when the spasm occurs; and it becomes so severe as to prevent sleep, and draw forth the most piercing cries: nevertheless, with the daylight relief from pain occurs, and few patients then retain any feeling of the past sufferings. The part when examined, either before or during the attack, does not exhibit any visible alteration, neither tumefaction nor heat, although the patient, while the pain endures, complains of this latter sensation, and throws off the bedclothes, or anything else that may tend to increase the feeling. Cold applications, such as plunging the part into cold water, cause the most acute suffering; which is also increased by moving the limb. An evident crepitation of the tendons is occasionally heard, resembling the sound emitted by walking on snow. One limb only is generally attacked; and women are more liable to it than men, especially robust women, between twenty and thirty years of age, whose occupations require fatiguing motions of the upper arms.

In consequence of the regular periodicity with which the disease returned each evening, M. Gambèrini was induced to exhibit the sulphate of quinine, but in vain. After trying several other remedies, he found that belladonna, made into an ointment as follows,—lard, ten drachms; extract of belladonna, four scruples, and used in frictions on the painful parts,—was possessed of the required efficacy. The general indications should be attended to at the same time: thus, in a strong, plethoric patient, in addition to the use of the belladonna, M. Gambèrini employed bleeding with advantage.—*Il Raccoglitore Medico.*

Paralysis independent of Lesion of the Nervous Centres.—Dr. Luyckx records the case of a woman sixty years of age, who, having been exposed to cold and wet for a long time, gradually lost the use of her legs, and afterwards of the arms. The limbs were in an edematous state, and cold. The intellect was unaffected, and sensation in the parts continued. The patient was treated by emetics, purgatives, blisters, &c. In ten days improvement commenced; and in less than three months she was cured. M. Luyckx considers that in this case the disease was in no way connected with the nervous centres, but was seated in the peripheric nerves.—*Annales de la Société de Médecine d'Anvers.*

Œsophageal Catheterism of the Insane.—The thesis of M. Emile Blanche on œsophageal catheterism of the insane is reviewed in *L'Union Médicale.* We extract from that notice the following article :—

"Esquirol was the first who proposed introducing through the nostrils into the œsophagus a gum-elastic catheter, in order to convey liquid food into the stomach. Two surgeons, Antoine Dubois and Murat, accordingly practised the operation, without in any way modifying the instrument which was used, although the route it had to traverse was very different in its anatomical aspect. The consequence was, that it did not always succeed; sometimes it was attended with considerable danger, and followed even by death. To remedy this, certain modifications in the instrument were made. The first and most important, by Dr. Ferrus and Dr. Mitivié, was the substitution of an ordinary gum-elastic catheter for the œsophageal instrument. This has been used successfully for five months together in one case by Dr. Ferrus; and for seven weeks at a time, by M. Blanche, on an insane patient at the Salpêtrière. The latter states that the operation with this instrument is easily performed, and with proper care we may be sure of succeeding. There are, however, certain difficulties connected with its use : when the catheter reaches perpendicularly the posterior paries of the pharynx, it may form an arch. If it be not very flexible, it curves with difficulty; and if force be used, this part of the operation may be painful. When the lunatic has acquired by practice the art of applying the base of the tongue against the posterior paries of the pharynx, by energetically contracting the muscles of that part, the obstacle to the passage of the catheter becomes serious. In such a case it may curve on the base of the tongue, and pass into the mouth, and then, by using a little force, a false passage may be made, by perforating the pharynx. The catheter may also pass into the larynx, or it may bend at the part where its eyes are situated, and thus form an obstacle which will prevent the passage of food, even when the instrument is in the œsophagus. To remedy these inconveniences, M. Baillarger provided the catheter with two stilets, one of steel, and the other of whalebone; the first, bent to a proper angle, enabled the operator to avoid the posterior paries of the pharynx; the second, after the first had been withdrawn, straightened the catheter by its elasticity, and applied it against the pharynx, thus withdrawing it from the glottis. The double object of the operator was thus answered; but it was attended with the difficulty of withdrawing the stilet in its curved state through the nostrils, the vertical diameter of which is not sufficient for that to be done readily; its extraction therefore causes a dragging and a compression which is always painful. To do away with this, M. Blanche has invented an articulated stilet, the rings being thirty-one in number, and so arranged as to play freely when flexed, but when extended, which is effected by a watch-spring working inside the rings, and soldered to a stiff and moveable stem, it assumes all the rigidity of a non-articulated stilet. This instrument, then, after it has been passed in the cnrved state through the nostrils into the pharynx, can be straightened, and passed on, so as to avoid the larynx. All that is requisite is to pull upon the watch-spring, which becomes straight, drawing with it the rings of the metallic chain, and thus apply the catheter against the posterior pharyngeal paries. When it has reached the œsophagus, the stem connected with the watch-spring is let go, the articulated part resumes its flexibility, and the stilet can thus readily be withdrawn."

This instrument appears to be a valuable modification and improvement on those previously in use.

M. Brierre de Boismont mentioned at a meeting of the Sociéte de Médécine at Paris, reported in the *Revue Médicale,* that in cases of insane persons who refuse to take food, he puts on the strait-waistcoat, and then introduces, by force, a catheter through the nose into the pharynx. He does not consider it requisite to pass it into the œsophagus. Some liquid aliment is then introduced through this canal; and he asserts that the agony the proceeding causes, as well as the imminent sense of suffocation, are such that the patient never forgets it, and will afterwards take the food that is offered to him, for fear of being again subjected to the same.

Medico-Legal Report on the State of Mind of Marie Madeleine Droin.—M. Girard has published in the last number of the *Annales Médico-Psychologiques,* a medico-legal report on the state of mind of a woman named Marie Madeleine Droin, accused of attempting to poison the daughter of a neighbour with whom she had been previously on good terms. It appeared that prior to this event she had manifested unequivocal signs of mental derangement, dating from the loss of an only child, about six years previously, to whom she was greatly attached. Her character changed; she became ill-tempered and malicious, rejoicing when any ill befel her neighbours; and instead of living comfortably with her husband, she made his life unhappy. Her mind was said to be constantly occupied and disturbed, without however being regarded as insane. Her whole pleasure seemed to consist of speaking of her lost child, except when she alluded to the misfortunes of others. The reason assigned for the attempt at poisoning the child was, that she might cause great grief to the mother, with whom she had had a quarrel. The witness by whom this opinion was advanced, stated that she had heard several persons ask if the prisoner's troubles had not affected her intellect, since she was continually speaking on that subject, prior to the attempt at poisoning.

It is further stated, that the prisoner scarcely ever is the first to speak; that she replies only when spoken to; that she is habitually sad, and very silent, remaining seated with her eyes cast down, passing her hand over her forehead; and not paying any attention to that which is going on around her, and that her appetite is disordered, she scarcely ever eating anything.—M. Girard asks, are not these the characteristic signs of melancholy insanity?

Other extravagant actions, committed before the crime of poisoning was attempted, are also brought forward, as for instance, the attempting to pull down the bed-curtains when in bed, cutting her own clothes and those of her deceased child to pieces, and making up her bed in the granary, &c. These attacks came on at the time when the catamenia were present. Some of the witnesses deposed to incoherence of speech on the part of the prisoner; and after she was confined in prison a short time, she complained of there being black cats in her room; she was constantly speaking of self-destruction. Her symptoms are thus summed up by the reporter, as indicative of melancholic insanity:—"Nothing is wanting," he says: "the attitude, gestures, physiognomy, want of sleep, loss of appetite, love of solitude; the delirium of the passion which is the strongest in women, the love of offspring; hallucinations, convulsive movements, extravagant ideas, represented by equally extravagant actions, having the character of melancholy, and extending as far as the manifestation of the desire for death, and finally, the predominance of a fixed idea, impressing its seal in the whole moral and physical being. The correctness of her memory as to dates, coherence in her conversation, reason in her actions, and the regularity of her vital functions, are not," says M. Girard, "incompatible with insanity."

The personal examination of the prisoner, by the reporter, confirmed these views. A well-marked expression of wandering and sadness was evident in the attitude and physiognomy; she appeared agitated and oppressed, complaining of want of appetite, thirst, headache, heat in the bowels, and constipation; this latter lasting for a week at a time. She thinks she sees her daughter, hears her voice; perceives flames; is insomnolent, and goes so far sometimes as to suppose that she embraces and touches her daughter, who suddenly disappears, so that she only seizes a shadow. She denies obstinately having given poison to her neighbour's child, but manifests great hatred against the father, who, she says, has several times attempted to seduce her. Her conversation does not betray any sign of general insanity, except when on the subject of her daughter, to which she constantly and irresistibly led it. The remembrance of her child caused her tears to flow, and gave a very singular convulsive expression to her face.

M. Girard drew the conclusions from the preceding facts, and from others which are detailed in his report, that the prisoner laboured under partial melancholic insanity, characterized further by a marked general disorder of the intellect at intervals only, and especially at the menstrual periods. He considered, therefore, that the attempt at poisoning was the result of feelings of hatred and vengeance, out of proportion to the causes which gave birth to them, caused by a perversion of the moral sensibility and of the intellect from partial melancholia.

The woman was acquitted on the ground of insanity.

Decrease of the Brain in Insanity.—From a paper read by M. Parchappe, before the Academy of Sciences, "*On the gradual decrease of the brain, in proportion to the successive degradation of the intellect in simple mania,*" we learn that since the publication of his treatise on insanity in 1841, in which he brought forward the preceding pathological law, the author has continued his researches, and has obtained a collection of 498 additional microscopic observations, confirming all the anatomico-pathological conclusions he had advanced. The existence of this pathological law of the gradual decrease of the brain in proportion to the successive degradation of the intellect springs from the comparison of the two categories, acute and chronic insanity, the medium of which differs in a quantity equal to eighty-nine scruples (grammes) in men, and eighty-five in women, in a proportion equal to 77·1000 in man, and 67·1000 in woman. It becomes still more evident, by the comparison of the medium in the four categories of chronic insanity, where we see the weight of the brain diminish at the same time with the intellectual power, and where the difference between the medium of acute insanity, and the last degree of chronic insanity, is as high as 152 scruples in man, and 135 scruples in women.—*Archives Génerales de Médécine.*

On Cerebral Congestion, considered in Relation to Hemorrhage and Softening of the Brain.—Dr. Durand-Fardel read a memoir on this subject, some months since, before the Academy of Medicine, an abstract of which is inserted in the *Archives Génerales de Médécine.* The two most common affections of the brain in the adult and in old age, hemorrhage and softening, cause, he says, organic lesions in the cure of which the vis medicatrix Naturæ plays the principal, if not the only part. Medicine can only be useful in seeking to prevent these diseases. With that object in view, we must become acquainted with the premonitory symptoms, and subject them to treatment as they occur. The precursory phenomena of hemorrhage and softening of the brain, when they exist and can be observed, are those indicative of cerebral congestion, such as headache, giddiness, numbness, and formication of the limbs, determination of blood to the head (*coup de sang*), &c. The different forms that acute softening may assume at the commencement—such as an attack of apoplexy, delirium, convulsions, and gradual and rapid abolition of the functions of relation—are identical with those which most frequently characterize cerebral congestion. In the body, the nearer the commencement of the disease, acute softening, is observed, the more certainly are traces discovered of general or partial hypertrophy of the substance of the brain. The more ordinary commencement of softening by an apoplectiform attack announces that the brain has been suddenly invaded by general hyperemia, to which succeeds the partial softening which is discovered at the autopsy, or which becomes chronic. With respect to hemorrhage, whatever part be ascribed to the alteration of the parietes of the vessels of the brain, which is common in old people, or to that lesion of the nervous pulp, called by M. Rochoux, "hémorrhagic ramolissement," the existence of which cannot be contested, at least in the greater number of cases, it is not possible to account for the hemorrhage itself, except by the existence of a determination of blood to the brain. We often find traces of this determination of blood in the bodies of persons deceased from hemorrhage, although the time that has elapsed, the treatment that has been adopted, and the unloading afforded by the rupture of the vessels appear sufficient to cause it to disappear. *En resumé:* if we ought not to award to cerebral congestion, or even to fluxion of blood, so direct a part in the preparation and production of these two diseases, it is impossible not to recognise a striking relation between them; whether the question be studied on the body, or during life. The disposition of the system of the encephalic circulation, the extreme vascularity of the brain, the size of its arteries, the extreme division of their branches, the obstacle to the return of the venous blood by the narrow and difficult openings in the cranium, explain the complicated *rôle* that cerebral congestion plays in the pathology of the brain. We may also readily find in it the explanation of a great number of cases of serous infiltration, so often met with in the subarachnoid cellular tissue.

Epileptiform Hysteria.—In *La Verdad*, a Spanish medical journal, we find the details of a case of epileptiform hysteria. The patient was a female servant, twenty years of age, unmarried, and of rather weak intellect. Epileptic symptoms first showed themselves during infancy, in consequence of blows on her head. They re-appeared at puberty, accompanied by cerebral inflammation, the result of injuries and attempts on her chastity. While residing in the house of Dr. Arguinosa, she complained one day of fatigue, and pain in the loins. The precursory symptoms of an epileptic attack soon showed themselves, and a few days afterwards, on returning from a short walk, she had scarcely time to throw herself on a bed before she lost both sensation and motion. Skin hot; respiration loud, and as frequent as the pulse; pupil immovable; eyelids closed convulsively; limbs flexible; while the lips were convulsively moved, or else a sardonic smile sat upon them. Bleeding was about to be practised, when all of a sudden, after some horripilations, the skin became cold and colourless; the pulse and respiration were suspended; and the insensibility and immobility were such, that had it not been for some slight nervous contractions, it would have been thought that the patient was dead. Affusions of cold water on the head seemed alone to produce any effect; the respiration then became agitated, the pulse strong, and violent convulsions, with tetanic rigidity (pleurosthotonos), set in. Dr. Arguinosa considered the case to be one of catalepsy, with nervous irritation of the spinal marrow. *Venesection; assafœtida injection.*

During the night a less severe degree of excitement alternated with well-marked collapse. The patient was then enveloped in a sheet soaked in warm vinegar-and-water, which was renewed thrice daily. This was attended with a beneficial result; the symptoms lessened in severity, and the next day the patient could express her desires by gestures, and reply to the questions which were put to her. A glass of orangeade, however, having been offered her, she upset it suddenly, screamed out, and fainted. All efforts to cause her to swallow liquids were afterwards useless. Liquids were not the only excitants of her anger and of her attacks; but the light also, the noise or the steps of persons around her, the unexpected presence of any one, and the slightest contact with her body or the curtains of her bed: the influence of my voice, however, says the author of the case, in withdrawing her from her attacks of delirium, and the preternatural reason and intelligence with which she expressed herself as soon as she recovered her speech (the second day), were very singular. She had a presentiment of her approaching death, and stated that she had never been bitten by any animal. The next two days there were the same alternations, the delirium occurring less frequently, and lasting a shorter time; she foamed at the mouth, when regurgitation, or hiccough, occurred. She slept but little that night (the fourth); the next day the only symptoms noticed were aversion to water, light, and air, with the pain of stomach previously complained of. The sixth day she asked for a bath, and the opium, which she took in the evening. A stool brought on strong convulsions and noisy delirium. The women who were attending to her believing her to be possessed by the devil, sprinkled her with holy water, which increased her furious cries and bizarre contortions. The following night was dreadful—the mouth full of foam, the eyes injected, and the delirium almost continuous. About two in the morning immoderate laughter succeeded the previous complaints. She then expressed her thanks and gratitude to Dr. Arginuosa, and shortly afterwards expired.

The editor considers that this singular disease was neither catalepsy, tetanus, hydrophobia, nor epilepsy, but epileptiform hysteria. To us it appears more to resemble hydrophobia than any other disease we are acquainted with.

Palsy of the Tongue Cured by Galvano-Puncture.—In 1813, an old woman, Rosa Ponti, was seized with general palsy of sensation and motion, in consequence of fright. Of this she was cured, except in the arms, head, and tongue. She could not articulate a word. In 1836, twenty-three years afterwards, Dr. Dacamina had recourse to galvano-puncture—one pole of the apparatus being applied to the occipital nerve by means of a needle introduced into the neck, and the other to the tip of the tongue. After two applications the patient could raise the organ, and after the third she could reply to some questions intelligibly, although with difficulty. After this the points of contact were varied, and the electricity applied to different parts. The patient gradually recovered her speech, and the other palsied parts were also cured.—*Filiatre Sebezio.*

Apparent Death.—In a memoir on *Apparent Death, and the Means of Preventing Premature Interment,* presented to the Academy of Sciences, by M. Bouchut, a candidate for the Manni prize, it is stated, that all apparent deaths, particularly those which are owing to asphyxia or syncope, present, whatever may be the diversity of their symptoms, one character in common—the *persistence of the pulsations of the heart* —a character which distinguishes them from real death.

The immediate and certain signs of death, according to M. Bouchut, are, the prolonged absence of the heart's pulsations, as ascertained by auscultation; the simultaneous relaxation of all the sphincters, owing to the paralysis of those muscles; and, finally, the sinking of the globe of the eye, and loss of the transparency of the cornea. The Commissioners of the Academy, Messrs. Dumeril, Andral, Magendie, Serres, and Rayer, admit the correctness only of the first of these: the others they look upon as doubtful. The certain signs of death, which occur at a later period,—cadaveric rigidity, absence of muscular contraction under the influence of galvanic stimulants, and putrefaction,—are admitted by all medico-logists. The Manni prize was unanimously awarded to M. Bouchut, as the author of the best memoir which has been sent in during the last ten years.—*Revue Médicale.*

INSANITY.—M. Bouchet, principal physician to the asylum and hospital at Nantes, has addressed a letter to the Academy of Medicine, in which he expresses his opinion that vice and crime are the consequences of moral diseases, which should be repressed for the preservation of society, and treated in appropriate penitentiaries. For such cases he disapproves of the Auburn system, and considers the Pennsylvanian plan, under proper restrictions, more adapted, and more likely to prove of service. Insanity affecting the intellect is ameliorated generally, he observes, by the patients mixing in society, subject, of course, to certain restrictions; general insanity, however, is rendered worse under the influence of those conditions which incessantly excite the sensibility, and turn it from its normal course.—*Revue Médicale.*

ACUTE MANIA.—A robust man was brought to the Bicêtre, and placed under the care of Dr. Delasiauve, for an attack of acute mania. His clothes were in rags, and covered with mud; his chest, knees, face, and hands bearing numerous traces of bruises and scratches. He did not present any symptoms of disease, other than the disordered state of the brain. He was bled, and put into a warm bath for two hours. For two days all went on well; afterwards the bruises and scratches became inflamed, and those on the hands suppurated freely. The wound made by the lancet also inflamed, and yielded some unhealthy pus. These symptoms were scarcely relieved before erysipelas attacked the face, part of the scalp, and the parotid regions, and was accompanied by great disturbance of the circulation, the pulse being small and irregular, and so frequent that it could hardly be counted. An emeto-cathartic and simple diluents removed the disease in about forty hours; but it was followed the succeeding morning by extensive meteorization of the abdomen, so as to raise the false ribs, and render respiration very difficult. The patient did not complain of pain; the only symptom he presented was the tympanitis. Magnesian water and astringent fomentations relieved him for a short time; but the meteorization soon reappeared, and was attended with a great discharge of blood with the urine. Attention was thus directed to the urinary passages, and it was not difficult to ascertain, by percussion and by palpation of the hypogastric region, that the bladder was distended by a liquid—a distention which, acting sympathetically on the intestines, and mechanically, by compressing them backwards, evidently contributed to the abdominal meteorization,

On the introduction of the catheter, a quantity of a thick, red liquid, exhaling a fetid ammoniacal odour, and composed almost entirely of blood, mixed with a certain proportion of pus, was drawn off. It appeared afterwards, on inquiry, that the man had long been subject to lumbar pains and hematuria.

The evacuation of the bladder afforded considerable relief for a time, and the tympanitic state of the abdomen diminished; but unfortunately the hemorrhage continued, and again distended the bladder, requiring the catheter to be used night and morning, a liquid precisely similar, and more or less highly coloured, being drawn off on each occasion. The man gradually sunk, and died a few days afterwards.

The examination of the body showed that the kidneys were greatly hypertrophied, softened, and injected; the right kidney contained a large quantity of black and half-coagulated blood, which was also found in the calices and pelvis. The mucous membrane was of a blackish, livid tint, and presented signs of ramollissement. The disease had proceeded to a greater extent in the left kidney. There was very little blood effused, but its place was supplied by a thick, reddish, purulent liquid, which poured out of the calices, and filled the pelvis of the organ. The kidneys presented traces of most intense inflammation; the mucous membrane was in some parts destroyed, and in others covered with pseudo-membranes. The disease extended along the ureters. The parietes of the bladder were greatly thickened, owing, apparently, to the hypertrophy and induration of the cellular tissue between the membranes. Its internal surface resembled that of an uterus affected with chronic metritis. On scraping it with the scalpel, a layer of detritus, consisting of mucus, pus, and decomposed blood, was removed, exposing beneath a very adherent, strong, and rough false membrane. This state of disease extended partly into the urethra, especially to the prostatic portion and the fossa navicularis. The membranous portion was in a state of ramollissement; the bulb hypertrophied. At that part the canal was greatly dilated.

The abdominal and thoracic viscera were healthy, with the exception of the liver, which was increased in size, and, by its granular and yellow appearance, evinced a tendency to the fatty degeneration. The ventricles of the brain contained a small quantity of reddish serosity, as did also the cavity of the arachnoid. There was also infiltration in the subarachnoid cellular tissue, and a well-marked injection of the pia mater, especially on the sides of the posterior lobes. The grey substance of the brain was slightly discoloured.

M. Delasiauve, the reporter of this case, was inclined to attribute disease to some syphilitic alteration, but he was unable to trace it the to that source. Compression of the vesiculæ seminales showed that, instead of the spermatic fluid which they ought to contain, they were full of an abundant matter having the colour and consistence of pus. He was of opinion that the mental disease, especially as it assumed the characters of melancholia, was brought on by the extensive disease discovered in the urinary organs.—*Revue Médicale.*

Epilepsy.—M. Péraire, considering epilepsy to depend on a momentary cerebral congestion, proposes to cure it by obliterating the numerous arterial branches which are distributed to the pericranium by subcutaneous incisions.—*Revue Médicale.*

Cold Affusion.—M. Baillarger communicated to the *Societé de Médécine de Paris,* the case of an insane lady he attended fifteen years ago, for whom he ordered cold affusions, which were effected by pouring one or two pailsful of water over the body. Following one of these affusions, his patient was seized with severe hæmoptysis, and died eighteen months afterwards from pulmonary phthisis.—*Revue Médicale.*

Treatment of Delirium Tremens.—M. Brierre de Boismont, in drawing the attention of the *Societé de Médécine de Paris* to the treatment of this disease, observes that the majority of practitioners have recourse to opium, exhibited by the mouth or in the form of enema; while others, again, have effected a cure by the abstraction of blood, and again in other cases by isolation alone, and momentary abstinence from strong liquors. M. Brierre de Boismont seeks to point out the cases in which venesection may be useful, and those in which it should not be practised.

When the patient is strong, plethoric, sanguine, and subject to congestions or *coups de sang,* he is of opinion that bleeding may be practised, but in moderation, its results being carefully watched. This practice appears to be more suited when the trembling is but slight, there are not any convulsive signs, and the incoherence is not strongly marked. He avoids bleeding when the patient is nervous; a state which may very well co-occur with all the appearances of a sanguine temperament, when the drunkard has been rarely or never bled. Those persons also in whom the delirium supervenes suddenly and violently, when in its symptoms it resembles ataxic meningitis, or in whom the trembling is well marked, or there are convulsive symptoms, great incoherence of speech, small pulse, or a haggard expression of the eyes, are not fit subjects for bleeding.—*Revue Médicale.*

Gastralgia and Hypochondriasis.—Under this title a case is recorded in the *Revue Médicale,* which appears to us to be one of the examples of spermatorrhœa. The patient drew up the details of his case himself.

*Physical Condition.*—Pain and burning, insupportable itching and tickling at the left side of the base of the cranium; the symptoms extending to the back and arm-pit of the same side; pains in the joints of the arms and legs; respiration difficult, oppressive; digestion painful; urine loaded; complexion yellow, earthy; eyes yellow, surrounded by a black circle; expression fixed; expectoration null; nasal secretion suppressed; pimples on the face, which is sometimes bloated, and sometimes extremely thin; limbs dragging; mouth pasty; loss of taste; numerous involuntary pollutions during sleep; and, lastly, general debility. The patient thought afterwards that he had cured himself of the pollutions by taking camphor in the morning fasting. The moral state was still more afflicting. A vague feeling of general suffering, experienced by the mind and soul more than by the body; profound sadness; utter depression; incapability to laugh or cry; avoidance of all society; desire for solitude; restlessness; agitated nights; insomnia; incubus; hallucinations; always discontented, never even a ray of pleasure; incapable of the least work, or at least of finishing that which had been begun; excessive slowness in the execution of all things; more or less loss of memory, and great idleness; in fine, taciturn and a dreamer, the patient passed all his time in pulling out the hair of his head and whiskers; and, a burden to himself, and doubtless to others, he constantly entertained the idea of suicide, which he was prevented carrying into effect by a religious feeling,

Bleedings, salt-water baths, travelling, magnetism, hydrotherapeia, and a host of nervous medicines, were tried in this case, but uselessly. Wearied by all this, the patient determined to treat himself. He commenced by taking an emeto-cathartic, which acted well; a brisk purgative was then had recourse to for several days, and afterwards a milder one substituted, until it was no longer needed, the cathartic being again employed when circumstances indicated the necessity. Depuratives, and bran and gelatine baths, at the temperature of from 72° to 74° F. were next used, with exercise on foot and horseback; music, both vocal and instrumental; abstinence from all spirituous liquors, pure, and especially white wine, coffee, tea, salted or highly-spiced meats, and, in fact, everything that might irritate the nerves. A mild regimen was observed; and all excesses avoided. This plan was pursued for six months, and now the patient says he enjoys excellent health, sleeps for six hours at a time; rises as soon as he wakes; and is as fresh and happy now as he was sad and depressed six months ago. He has regained the integrity of his intellect, and can hear and understand well; and his memory is excellent.

THE ORGANIZATION OF LABOUR FOR THE INSANE.—M. Parchappe observes, that if the utility of labour in lunatic asyla were confined to its curative influence, its importance would be greatly diminished, for the patients who are really curable constitute but a very small proportion of the population of these establishments. But labour is in lunatic asyla, as in all large collections of men, an essential condition for the maintenance of order, and the preservation of good conduct. The well being of incurable patients even is not less intimately connected than that of other men with the laws of labour, whether it be considered as an hygienic measure for the preservation of health, by the maintenance of the equilibrum of the strength, or as a moral power capable of keeping the mind at peace, by the removal of sorrow and ennui.

It is evident, therefore, that as the greater number of insane patients in asyla are incurable, and as the latter only can be engaged in continuous labour, and that for a long time, it becomes important, in the organization of such labour, that it be adapted to the condition and wants of the incurables. In the first place, the occupations should be healthy, agreeable, and productive. In point of salubrity those occupations which bring all the muscles into action, and which necessitate exercise in the open air, are those which should be preferred. Sedentary occupations, which alone are adapted for a great number of women, can hardly be considered healthy, unless interrupted from time to time by intervals of sufficient length, during which exercise may be freely taken in the open air. They should be pursued in large well-lighted and well-ventilated rooms, free from the extremes of heat and of cold. Labour may be made attractive, by varying the occupation, by changing the place where it is carried on, and by the satisfaction experienced by the creation of the products, and still more by the predilection for an occupation which the patient may have chosen, and to which he is habituated. It is therefore important, in allotting work for incurable patients, to select such as may the nearest resemble their employments before they were admitted into the asylum. The productiveness of the labour will be regulated by the wants of the asylum. The first step in this respect would be the supply of the asylum itself with whatever may be required by the work of the lunatics, and thus render it independent of out-door labour. Should there not be sufficient occupation thus afforded, other work may be given, regulated according to the predominating character of the industry of the country. Thus, in the asylum at Ghent, the fabrication of lace by the female patients is recommended, and the culture of silk-worms in the South of France.—*Annales Medico-Psychologiques.*

Acute Tubercular Meningitis in the Adult.—M. Levy, principal physician in the hospital Val de Grace, has reported three cases of this disease. In the first, the tubercular affection of the pia mater was complicated by softening of the brain, tubercles in the mesenteric and bronchial glands, pneumonia, with pulmonary apoplexy, tubercular pericarditis, &c. The patient was twenty-six years of age. In the second case, besides the tubercular disease, there were, meningitis, softening of the brain, and tubercles in the lungs, kidneys, liver, and several other organs. The third case was an example of tubercular meningitis, developed as a sequence of measles, accompanied by chronic pleurisy, and preceded doubtless by numerous tubercles on the lungs, and the general tubercular diathesis.—*Bulletin de l'Académie Nationale de Médecine.*

Complete Paraplegia cured by Cold Bathing and Urtication.—Dr. Van Baugevem has published, in the *Annales de la Societé de Médecine d'Anvers,* the particulars of a case of paraplegia, complete as to sensation and motion, occurring in a scrofulous subject, from constant exposure to the vicissitudes of the weather. Frictions with camphorated spirit, blisters, and purgatives, were tried for five weeks, but having altogether failed, Dr. Van Baugevem caused his patient to be plunged every evening up to the middle in cold water, dry friction with flannel being practised afterwards, for half an hour, and then urtication. At the end of a fortnight there was considerable improvement; the flogging with nettles was then omitted, the cold bathing and the frictions with flannel only being persisted in. Three months afterwards the cure was complete.

Chloroform in Tetanus.—A young man of good constitution was admitted into the hospital, under M. Forget, labouring under acute idiopathic tetanus, the result of exposure to cold, while perspiring freely. The symptoms he presented, on admission, were those of incomplete trismus and opisthotonos. Bleeding and the internal exhibition of ammonia were at first tried, but without benefit. M. Forget then determined to have recourse to chloroform, four scruples of which, poured on a compress, were applied to the nose and mouth of the patient. From the increase of the general spasms, the moaning, the convulsive contractions of the respiratory muscles, and the turgescence and lividity of the face, it was feared that asphyxia was impending; the pulse, however, remained firm at 86, the respiration became stertorous, the foam which filled the mouth was cleared away, and the compress re-applied; all of a sudden the muscles became relaxed, the abdomen supple, the limbs fell lifeless, as it were, the relaxation and insensibility became complete, the colour returned, the stertor ceased, and a deep calm sleep supervened. The application of chloroform lasted from one to two minutes; the sleep continued about ten. The tetanic symptoms returned when he awoke. With the view of breaking the chain of morbid habit, it was determined to re-apply the chloroform, but only twice a day, fearing that if more frequently used, disease might be lighted up in the lungs. The same effects were constantly produced by the anæsthetic agent, the period of excitement, however, diminishing in duration, and that of collapse occurring more or less quickly. M. Forget mentions particularly the occurrence of well-marked divergent strabismus, as an indication that muscular relaxation was taking place. Under this medication, the disease slowly but surely amended; after the lapse of seventeen days from the first use of the chloroform, and thirty-four from the commencement of the disease, all tetanic symptoms had disappeared, and the patient was able to walk about—in fact, was quite convalescent. The strabismus mentioned as an indication of approaching muscular relaxation, it appears, was a condition of the rectus muscle of the eye, prior to the invasion of the disease; the squint, however, disappearing during the muscular contractions incident to the complaint.—*Bulletin Géněral de Thérapeutique.*

Melancholy cured by severe Bodily Injuries.—Dr. Labruyère narrates the case of a man, thirty years of age, who had been subject to melancholia (*délire melancholique*) for several years, and who was severely wounded by machinery, either accidentally or otherwise. He had extensive wounds of the face and cranium, the bones of the latter being broken, and the brain partially exposed; the forearm was also torn off. The treatment was necessarily protracted, but as cicatrization made progress, the complete cessation of the mental disease occurred.—*Journal des Connaissances Medico-Chirurgicales.*

Musk and Blisters in Acute Hydrocephalus.—M. Legroux, a physician practising at Beaujon, states that he has derived great benefit from the repetition of blisters and the exhibition of musk in the treatment of acute affections of the brain, when the following symptoms are present: coma, dilatation of the pupils, convulsive movements, and more or less extensive paralysis. He narrates cases in illustration of his opinion, and considers that musk acts by lowering the pulse, and the temperature of the skin, and by causing a velvety feel and relaxation of the integuments. In one case it acted as a sudorific. He considers musk to be a cardiaco-vascular hyposthenic, and perhaps also an hyposthenic of the nervous system.—*Bulletin Géněral de Thérapeutique.*

Mercurial Frictions in Encephalitis.—Dr. Privat, of Campagnac, employs mercurial frictions in the commencement of encephalitis, and when the inflammatory state is decreasing, but not when the disease is fully developed. Mercury is, he says, a powerful sedative of the nervous system, in cases of irritation and phlogosis, and a special excitant of the exhalent and absorbent system.—*Bulletin Général de Thérapeutique.*

Intermittent Cephalea caused by an Effusion of Blood between the Dura Mater and its Parietal Arachnoid.—M. Dubois, of Neufchatel, was called to see a powerful robust man, fifty-seven years of age, who complained of gastric disorder and intense cephalea, caused apparently by grief for the loss of his child. The headaches, which were very intense, proved to be intermittent. He survived about six weeks, dying comatose. The head was examined thirty hours after death. On the removal of the upper part of the cranium, a considerable effusion of a black liquid was discovered over the right hemisphere under the dura mater, or rather, the dura mater properly so called, and its parietal arachnoid layer. The cavity, which contained about half a glassful of the liquid, was of an oval shape. There was not any clot. The cerebral substance was not apparently altered, neither softened, nor injected, nor adherent to the pia mater, nor to the internal layer of the arachnoid, nor was the arachnoid sac thickened in any way, but smooth and shining, except at the adherences of the falx. A small effusion of coagulated blood, extending transversely, was found in the lower third of the vertical thickness of the tuber annulare. M. Dubois attributes the intermittent cephalea to the hemispheric effusion, and the final attack of coma to the collection of blood found in the tuber annulare. — *Bulletin Général de Thérapeutique.*

Aldehide.—M. Poggiale, professor of chemistry at Val de Grâce, states that the inhalation of the vapour of aldehide is rapidly followed by the most complete insensibility; its stupefying action is more rapid and more energetic than that of ether and chloroform. Its odour is very powerful, or otherwise it would be preferable to chloroform in an economic point of view. A very considerable quantity may be obtained by a simple operation; all that is requisite is to distil a mixture of sulphuric ether, water, alcohol, and peroxide of manganese, and to rectify the condensed liquid with chlorine of calcium. Aldehide thus prepared boils at the temperature of from 82 to 84 Fahrenheit, and contains very little alcohol and formic ether. Perfectly pure aldehide is not necessary.—*Académie des Sciences.*

Action of Cannabis Indica.—M. Courtive observes, as the result of numerous experiments made to determine the physiological action of the cannabis indica on the nervous system, that cannabine (the resin of the Indian hemp) may be useful therapeutically, as narcotic and stupefying, in the treatment of the neuroses in general, and in the last stages of cancerous diseases. Cannabine produces also tetanic effects, and in certain periods of its action it seems to be a general stimulant. In some cases haschisch causes sanguineous congestion of the lungs, which, however, M. Courtive does not consider a reason for rejecting it, as the congestion does not always occur, and may be removed by bleeding. It has been employed with advantage in some cases of hooping cough and bronchial catarrhs, and, according to M. Moreau, may be useful in mental pathology.—*Académie des Sciences.*

Periodic Epilepsy, Hysteria, Neuralgia, and Hemiplegia.—M. Mazade, in a communication made to the Academy of Medicine, M. Bricheteau, reporter, narrates cases in which the periodicity of these diseases was sufficiently marked to warrant the exhibition of the sulphate of quinine in rather large doses. The cases were successfully treated. The fifth case is described as a sort of hemiplegia which returned every day, that is to say, it was a momentary cephalic congestion, with notable numbness and weakness of the limbs of the right side of the body. This singular affection yielded to the employment of the sulphate of quinine for three days, in the dose of a scruple and a half.

Chorea in Scrofulous Subjects.—M. Muller describes two cases of chorea occurring in young girls of a scrofulous constitution, which were cured by the internal exhibition of the ioduret of potassium. Duration of treatment: in one case, thirty days; in the other, twenty-two. In the one case, thirty-two scruples of the ioduret were given; in the other, nineteen.—*Académie de Médecine.*

Influence of the Penitentiary System on Insanity.—M. Bouchet, principal physician to the Lunatic Asylum at Nantes, states, in a letter addressed to the Academy of Medicine at Paris, that in August, 1845, he received 15 lunatics from the prison at Vannes, of whom nine have been discharged, one died, and five are still at the asylum. Of the nine discharged, three were transferred, uncured, to the asyla of their departments, when the period of their imprisonment had expired; one was imbecile and epileptic; another laboured under old monomania, with hallucinations of the sight and hearing; but M. Bouchat cannot affirm that they existed before the imprisonment; the last was affected with that kind of reasoning or instinctive monomania, which, taking its origin in a lesion of the sensibility, does not sufficiently impair the reason to warrant the term insanity, until characterized by certain acts. Of the six others, three were affected with monomania, and hallucinations of the senses referrible to fear; these were discharged cured. The other three, one of whom was of weak intellect, also laboured under reasoning or instinctive monomania, of long duration, and before trial. Having become quiet and working, they were discharged; but one of them was afterwards arrested, and placed in the asylum belonging to her department. The patient that died was always weeping, in which state she was reported to have continued for ten years, although she was condemned in 1843 only. She believed her husband and children were dead, which was not the case. Of the five still in the asylum, the first, affected with monomania and hallucinations of sight, had been mad three or four years before she was tried; she had left her husband and children, to wander about the country, and in a moment, as she said, *when it was stronger than she was,* had set a house on fire. The second, two years before she committed the theft for which she was condemned, had been confined for eighteen months at Quimper, or at Morlaix, as insane; after being in prison four years, she complained of headaches, talked to herself, was annoyed at the slightest contradiction, and stabbed herself in the abdomen, because she was put in a cell. Hers was also a case of instinctive monomania, with well-marked intellectual debility. The fourth, condemned in 1844, had for twelve years heard voices, to which she replied, and which incessantly spoke to her of angels, the cross, &c. The fourth, also instinctive monomania, was an instance of great intellectual debility, and the fifth was a somewhat similar case; the patients, however, worked pretty well, except when inordinate and even violent instinctive acts occurred.

M. Bouchet, from these cases, cannot come to the absolute conclusion, that the penitentiary system was the essential cause of the insanity, which, in many cases, he observes, existed before the crime was committed for which the imprisonment was inflicted, and even was the determining cause of the crime, without evincing itself to justice by irrefragable evidence. No cases of mania, properly so called, presented under the circumstances.

The prison from which these lunatics were sent to the asylum at Nantes, is conducted on the Auburn system.

Eclampsia cured by Stramonium.—Dr. Armand Jobert narrates the case of a young idiot affected with eclampsia, which, after resisting all the usual remedies, was cured by accidental poisoning by stramonium, the patient having found and eaten five of the thorn apples. Dr. Jobert is unable to say whether the cure was permanent, as the patient afterwards passed from under his care. The idiocy depended on a physical defect of the brain.—*Annales Medico-Psychologiques.*

Tetanus Cured by Intoxication. — In the same journal, Dr. Jobert describes a case of tetanus occurring in a child five years old from a cut of the finger, which he cured by intoxicating the child. The patient became quite drunk and delirious; vomiting, diarrhea, and general prostration, sweating, and deep sleep, followed. The next day, the alarming symptoms of tetanus were removed, and the child soon became convalescent. Leeches were applied behind the ears, to relieve a determination of blood to the head, the result of the intoxication. A similar case, with the same result, was recorded in one of the French journals last year, and another case, in which the treatment was much more protracted, has been read at a meeting of the Medico-Chirurgical Society. In a case of hydrophobia, which, however, terminated fatally, Mr. Fitzpatrick afforded great relief by the same measure.

Effect of Music on the Nerves.—The first effect that music produces is merely physical. Sounds strike the nerves of the ear, and make, according to their power, quality, character, roughness, or sweetness, analogous impressions upon our senses. Too powerful, too sudden strokes, might occasion nervous convulsions, destroy the faculty of hearing, or even extinguish life. In the endless variety of organizations, an endless variety of sensations is awakened by one and the same sound. A delicate, sensitive ear, is otherwise affected than that of a stronger, rougher nature. The tumult, the whistles, and the shrieks, in which the latter delight, would throw the other into the highest state of alarm or discomfort. Hence the curious anecdotes of men who could not tolerate certain sounds, and who, on hearing particular instruments, became the subject of the most unaccountable nervous sensations, of which no other person was conscious. The beating of a drum produces in many persons a corresponding tremulousness in the chest. Some persons cannot refrain from weeping when they hear a certain note. J. J. Rousseau says that he knew a lady who could not hear any kind of music without being seized with involuntary and convulsive laughter. The delicacy of the ear of Mozart was so great, that, without being modified by other instruments, he could not tolerate the sound of a trumpet. His father, who wished him to overcome this sensibility, took him one day by surprise with a violent blast of a trumpet. The boy shrieked, grew pale, and fell senseless to the ground. The monk of St. Gallen tells us of a woman, who, when she heard an organ for the first time, was so transported with rapture, that she never recovered from the effect, and died in consequence. Animals, as well as men, are accessible to the effect of sound. The musical susceptibility of certain dogs is very remarkable. In the year 1827, all Rome went to see a dog who every day marched at the parade before the military band; in the evening, he was found sitting in the orchestra of the Theatre Della Valle; no one ever knew whence he came, or to whom he belonged. But sounds not only act upon the nerves of man and animals, but even upon inanimate objects. Glasses, mirrors, china vases, have been seen to vibrate and break at certain sounds of the flute, and notes of the human voice. We may witness the latter in cathedrals, where certain pipes of the organ cause a shaking of the windows, walls, and pillars. A powerful bass voice, in the lower notes, has, in a limited space, the same effects upon chairs and tables. We see, from these simple facts, that the magical power of music is not always attributed to its right cause. We are too often inclined to ascribe to the talent, either of the composer or of the performer, the whole merit of the wonderful effects produced. Undoubtedly, the power of talent and genius, in the melodious and harmonious combinations of sound, should be acknowledged; but it should not be forgotten that there is, in the nature of sound itself, something mysteriously affecting. A simple sound, by whatever physical cause produced, similar to that which issued from Memnon's statue, or which is heard from Fingal's cave, at Staffa, excites in us, with all its monotonous repetition, a something that either fills us with melancholy or with awe. At midnight, the repeated call of the sailor who sounds the depth, or the blast of a trumpet heard from a watch-tower or minaret, has in it something solemn and majestic. —*Dr. Mainzer's Music and Education.*

